# How to Prevent Colds

**Published:** 1876-05

**Authors:** 


### HOW TO PREVENT COLDS.

If people were blessed with common sense, and a little wholesome self-denial, they might often escape severe colds and fevers by resolute measures adopted in season. A correspondent of the *Evangelist* sends the following communication, giving an infallible recipe for a bad cold, if it is handled in time. Perhaps some of our readers may have courage to make the experiment:

"There is probably not a man, woman, or child, who is not as often as once a year afflicted with a severe cold, which ends in a cough or catarrh; and thousands there are who die every year of consumption, brought on by taking cold. He, then, who should discover a certain and effectual remedy for this complaint would be justly regarded as one of the greatest benefactors of the age. The writer does not profess to have discovered such a remedy, but he wishes to attest the truth of the following certain and effectual expedient for preventing a cold. A cold cannot be easily cured; but if it can be prevented, it is of no importance to know how it may be cured.

"A bad cold, like measles or mumps, or other similar ailments, will run its course of about ten days, in spite of what may be done for it, unless remedial means are employed within forty-eight hours of its inception. Many a useful life may be spared to be increasingly useful, by cutting a cold short off in the following safe and simple manner. On the first day of taking a cold, there is a very unpleasant sensation of chilliness. The moment you observe this, go to your room and stay there. Keep it at such a temperature as will entirely prevent this chilly feeling, even if it requires 100 degrees of Fahrenheit.

"In addition to this, put your feet in water half-leg deep, as hot as you can bear it, adding hot water from time to time, for a quarter of an hour, so that the water shall be hotter when you take your feet out, than when you put them in. Then dry them thoroughly, and put on thick, warm, woollen stockings, even if it be summer—for summer colds are more dangerous—and for twenty-four hours eat not an atom of food, but drink as largely as you desire of any kind of warm tea, and at the end of that time the cold will be entirely broken without any medicine whatever. Efficient as the above means are, not one in a thousand attends to them; led on, as most men are, by the hope that a cold will pass away of itself. Nevertheless, this article will now and then pass under the eye of a wise man who does not choose to run the double risk of taking physic and dying too."—*Medical Journal.*

"The above expedient is a severe one for epicures and gluttons, but most persons will find it easier to fast one day than to be sick a fortnight. The writer has usually found that fasting for three or four meals is sufficient; but doubtless the whole remedy is better than a part.

"Let those who are often afflicted with colds—ministers, students, and consumptives generally, cut out the above directions and preserve them; if faithfully followed, they will do you more good than all the pulmonaries, cold cordials, and other hurtful nostrums, which now load your shelves.—*Watch. & Refl.*

The above was sent to that excellent religious paper "*The Watchman and Reflector*," of Boston, by one of its correspondents, as having been taken from a "*Medical Journal*" Its intrinsic importance, its truthfulness, and practical utility merit a republication every year, in our pages where it first appeared.

